# Malaria Parasite CLAG3, a Protein Linked to Nutrient Channels, Participates in High Molecular Weight Membrane-Associated Complexes in the Infected Erythrocyte

**DOI:** 10.1371/journal.pone.0157390

**Published:** 2016-06-14

**Authors:** Kayvan Zainabadi

**Affiliations:** Laboratory of Malaria and Vector Research, National Institute of Allergy and Infectious Diseases, NIH, Rockville, Maryland, United States of America; Bernhard Nocht Institute for Tropical Medicine, GERMANY

## Abstract

Malaria infected erythrocytes show increased permeability to a number of solutes important for parasite growth as mediated by the Plasmodial Surface Anion Channel (PSAC). The *P*. *falciparum clag3* genes have recently been identified as key determinants of PSAC, though exactly how they contribute to channel function and whether additional host/parasite proteins are required remain unknown. To begin to answer these questions, I have taken a biochemical approach. Here I have used an epitope-tagged CLAG3 parasite to perform co-immunoprecipitation experiments using membrane fractions of infected erythrocytes. Native PAGE and mass spectrometry studies reveal that CLAG3 participate in at least three different high molecular weight complexes: a ~720kDa complex consisting of CLAG3, RHOPH2 and RHOPH3; a ~620kDa complex consisting of CLAG3 and RHOPH2; and a ~480kDa complex composed solely of CLAG3. Importantly, these complexes can be found throughout the parasite lifecycle but are absent in untransfected controls. Extracellular biotin labeling and protease susceptibility studies localize the 480kDa complex to the erythrocyte membrane. This complex, likely composed of a homo-oligomer of 160kDa CLAG3, may represent a functional subunit, possibly the pore, of PSAC.

## Introduction

Following invasion, the malaria parasite increases permeability of the infected erythrocyte to a number of key nutrients [1–4]. This increase in permeability, which occurs typically 12–16 hours post-invasion, is accomplished via the Plasmodial Surface Anion Channel (PSAC) [5]. PSAC is an unusual channel in that it shares little similarity with other channels and allows permeability of a wide array of solutes while exquisitely precluding sodium, thus preventing osmotic lysis [6]. Importantly, channel activity is essential for parasite viability: inhibition of PSAC with small molecules is lethal [7]. The molecular components of this channel were unknown until recently when parasite-encoded *clag3* genes (*clag 3*.*1 and clag 3*.*2)* were shown to be key determinants of PSAC activity [8, 9].

*Clag3* is a member of the multigene CLAG family consisting of c*lag2*, *clag3*.*1*, *clag 3*.*2*, *clag8*, and *clag9* [10]. There are now multiple lines of evidence implicating CLAG3 as a key contributor to PSAC function. First, a screen for channel inhibitors with differential effects on HB3 and DD2 identified *clag3* as the gene responsible for the observed phenotype [8]. A second independent screen using protease-induced channel inhibition as a phenotype again directly implicated the *clag3* genes [9]. Additionally, epigenetic silencing/switching and a point mutation in *clag3* have been associated with reduced uptake—and resistance—to the PSAC permeable antimalarial toxins, blasticidin and leupeptin [8, 11, 12]. Interestingly, epigenetic silencing of *clag2* was also associated with resistance, suggesting other CLAG family members may also contribute to PSAC function [11].

The role of CLAG3 in channel function was initially a surprise owing to its previous involvement as a member of the rhoptry-associated RhopH complex. Seminal studies demonstrated that CLAG3 (also known as RHOPH1) interacts with RHOPH2 and RHOPH3 in the rhoptries of merozoites, and that this complex is injected upon invasion [13, 14]. Afterwards, the proteins can be found at the parasitophorous vacuole membrane (PVM) and Maurer’s cleft of the newly invaded cell, as well as the extracellular medium [15, 16]. Importantly, attempts to knockout RHOPH2 and RHOPH3 have been unsuccessful indicating they likely serve an essential function that has yet to be elucidated [17]. While knockout data does not currently exist for CLAG3, epigenetic silencing of the gene has been associated with significant growth inhibition [18].

Although atypical for an anion channel, CLAG3 nonetheless has a number of properties consistent with a role in channel function. For one, biochemical experiments have shown that CLAG3 occurs both as an integral and peripheral membrane protein in the infected erythrocyte [8]. The integral fraction is susceptible to extracellular protease cleavage and thus localizes to the erythrocyte surface [8, 9]. In fact, CLAG3 contains an exposed hypervariable domain flanked by at least two putative transmembrane domains. One of these transmembrane domains is lined with polar amino acids on one side and non-polar on the other, reminiscent of a water-filled pore [19]. Intriguingly, the mutation in the aforementioned leupeptin-resistant parasite maps to this pore-like region [8, 19]. It is currently a mystery what role the peripheral fraction of CLAG3 plays in channel function or broader parasite biology.

While CLAG3 clearly contributes to PSAC function, exactly how it does so has remained elusive. As a result, many outstanding questions still remain: Is CLAG3 sufficient by itself to form the channel or are other host/parasite proteins involved? Does CLAG3 exist at the host membrane as a monomer or multimer? And why exactly does it take ~12+ hours for the channel to become active in the infected erythrocyte?

To begin to answer these questions, I have taken a classic biochemical approach. Performing immunoprecipitation experiments, I find that CLAG3 participates in at least three different high-molecular weight complexes, the smallest of which (480kDa) localizes to the erythrocyte membrane. This complex, composed solely of 160kDa CLAG3, represents an attractive candidate as the putative pore of PSAC.

## Materials and Methods

### Parasite Culture

*P*. *falciparum* laboratory lines were grown in RPMI 1640 supplemented with 25 mM HEPES, 50 μg/mL hypoxanthine (KD Medical), 0.5% Albumax (Invitrogen), 0.23% sodium bicarbonate (Gibco) using O^+^ human erythrocytes (Interstate Blood Bank) and maintained under 5% O_2_, 5% CO_2_, 90% N_2_ at 37°C. A recombinant HB3 parasite with a C-terminal FLAG-tagged CLAG3 (HB3^3rec^) as previously described was used for immunoprecipitation (IP) experiments [8]; an isogenic untagged HB3 parasite was used as a negative control.

### Immunoprecipitation Experiments

Membrane fractions were isolated as previously described [8, 9]. Briefly, percol-sorbitol enriched trophozoite-infected erythrocytes were incubated at room temperature with 40 volumes of hypotonic lysis buffer (7.5mM Na_2_HPO_4_, 1mM EDTA, pH 7.5 with protease inhibitors) for 5 minutes and then subjected to a 100,000x*g* spin for 1 hour at 4°C to pellet membranes. Where indicated, peripheral proteins were liberated from membranes by treatment with 100mM Na_2_CO_3_ pH 11 at 4°C for 30 min, followed by a second 100,000x*g* spin to re-pellet membranes. Membrane pellets were then resuspended (with vigorous pipetting) in lysis buffer containing 10mM Tris-HCl pH 7.5, 1mM EDTA, 150mM NaCl with protease inhibitors and appropriate detergent at indicated concentrations (w/v). Lysates were incubated with rotation at 4°C for 1 hour, spun at 100,000x*g* for 1 hour or 16,000x*g* for 15 minutes (where indicated) to pellet insoluble material, and the resulting supernatant was used for immunoprecipitation experiments.

Immunoprecipitations were performed with 50μl of FLAG-agarose (Sigma Aldrich) or NeutrAvidin-agarose (Thermo Scientific) for 1 hour at 4°C with rotation, spun down at low speed, washed 3 times with lysis buffer, and finally eluted with FLAG peptide (Sigma Aldrich) or cleavage of the biotin spacer arm with 100mM dithiothreitol (DTT) (Sigma Aldrich).

### SDS and Native PAGE Experiments

SDS PAGE was performed using 4–15% TGX gradient gels (Bio-Rad) according to manufacturer’s instructions. Lysates were mixed with NuPAGE dye (Invitrogen) to a final concentration of 4% LDS (lithium dodecyl sulfate) and 2.5% 2-mercaptoethanol, and separated at room temperature for 90 minutes at 150 volts. Native gels were run using the Blue Native PAGE system (Thermo Scientific) using Novex 3–12% Bis-Tris gels and the XCell SureLock Mini Electrophoresis System (Thermo Scientific). IP eluates were mixed with appropriate volumes of NativePAGE 4X Sample Buffer containing G-250 Sample Additive (Thermo Scientific) and separated at 150 volts for 1 hour and then 250 volts for 1 additional hour all at 4°C. Molecular weights were determined by use of the manufacturer provided native marker (Thermo Scientific) and confirmed by use of a second independent native marker (GE Healthcare). Gels were stained with either Imperial Protein Stain (Thermo Scientific) or Pierce Silver Stain Kit (Thermo Scientific) according to manufacturer’s instructions.

For immunoblotting, SDS PAGE gels were transferred to nitrocellulose membranes as previously described [19], and Native PAGE gels were transferred to PVDF membranes according to manufacturer’s instructions (Thermo Scientific). Blots were blocked with 3% milk in TBST (Tris-buffered saline with 0.1% Tween) (Sigma Aldrich), incubated with 1/2,000 dilution of FLAG (Sigma Aldrich); 1/1,000 dilution of biotin antibody (Cell Signaling); or 1/3,000 dilution of CLAG3 and RHOPH2 antibody, 1/1,000 dilution of RHOPH3 antibody (all obtained using a recombinant C-terminal fragment as described previously [8]). After incubation with primary antibody for 1 hour, blots were washed and detected with a horseradish peroxidase-conjugated secondary antibody (Pierce) using a chemiluminescent substrate (SuperSignal West Pico, Pierce).

### Cross-linking Experiments

Freshly made amine-reactive N-hydroxysulfosuccinimide esters (NHS-esters, Thermo Scientific) were added to lysates of hypotonically lysed cells at varying concentrations (0–500μm) according to manufacturer’s instructions. Cross-linking was carried out with gentle agitation at room temperature for 30 minutes, and then quenched with addition of 25mM Tris. Lysates were then spun at 100,000x*g* spin to pellet membranes before proceeding with the standard IP protocol as described above. To reverse cross-linking, Dithiobis (succinimidyl propionate) (DSP) cross-linked IP eluates were incubated with 100mM DTT for 30 minutes at room temperature.

### Extracellular Biotin Labeling and Protease Susceptibility Studies

Percol-sorbitol purified trophozoite-infected erythrocytes were resuspended in 40 volumes of PBS buffer with or without 100nm of PSAC inhibitor, MBX2366 (Microbiotix) [6]. Freshly made succinimidyl-6-(biotinamido)-6-hexanamido hexanoate (NHS-LC-LC-Biotin; Thermo Scientific) was then added at varying concentrations (0–250μm) and cells were allowed to label for 20 minutes at room temperature with gentle agitation. The reaction was quenched with 25mM Tris and cells were washed extensively with TBS. Labeling of intracellular proteins was ascertained by probing the ‘soluble fraction’ of hypotonically lysed cells (ie the supernatant following a 100,000x*g* spin) for biotin by Western blot.

Extracellular protease experiments were done in a similar fashion except cells were resuspended in PBS containing 0.6mM CaCl_2_ and 1mM MgCl_2_ and incubated with 1mg/ml pronase-E (a broad-spectrum protease mixture) (Sigma Aldrich) for 1 hour at 37°C. Reactions were terminated by addition PBS containing 5mM EDTA and protease inhibitors. Cleavage of intracellular proteins was assessed by coomassie staining of the total intracellular soluble protein fraction obtained as described above.

### Time-course Experiments

Parasites were synchronized by two subsequent treatments of 5% sorbitol separated by 8 hours. The following day mature schizonts were percol-sorbitol enriched and mixed with fresh (naïve) red blood cells to allow for reinvasion. After 6 hours (or 12 hours for cross-linking experiments) cells were synchronized with 5% sorbitol to lyse any unruptured schizonts and the resulting rings were harvested (without enrichment) for IP experiments at the indicated timepoints.

### Mass Spectrometry and Proteomics

Identification of gel separated proteins was performed on reduced and alkylated, trypsin digested samples. The supernatant and two washes (5% formic acid in 50% acetonitrile) of the gel digests were pooled and concentrated by speed vac (Labconco). The recovered peptides were re-suspended in 5μl of Solvent A (0.1% formic acid, 2% acetonitrile, and 97.9% water).

Prior to mass spectrometry analysis the re-suspended peptides were chromatographed directly on column, without trap clean-up. The bound peptides were separated using an AQ C18 reverse phase media packed in a pulled tip, nano-chromatography column from Precision Capillary Columns. The chromatography was performed in-line with an LTQ-Velos Orbitrap mass spectrometer (ThermoFisher Scientific) and the mobile phase consisted of a linear gradient prepared from Solvent A and Solvent B (0.1% formic acid, 2% water, and 97.9% acetonitrile) at room temperature. Nano LC-MS (LC-MS/MS) was performed with a ProXeon Easy-nLC II multi-dimensional liquid chromatograph and temperature controlled Ion Max Nanospray source (ThermoFisher Scientific) in-line with the LTQ-Velos Orbitrap mass spectrometer.

All searches were performed using PEAKS v7.5 (Bioinformatics Solutions Inc) against a custom database containing *Plasmodium falciparum* 3D7 proteins from the PlasmoDB (v27, 3/2016), Clag 3.1 and 3.2 sequences corresponding to HB3^3REC^ (Clag3.1 and 3.2 proteins from the PlasmoDB were removed from the search database for the purposes of this project) and common contaminant proteins from theGPM (cRAP.fasta, 1/2015). Peptides were filtered with a 0.5% FDR using a decoy database approach, and protein assignments required at least 1 unique peptide and 2 total peptides per protein. Laboratory and dust/contact proteins identified by the cRAP contaminant database were removed from the table for clarity.

## Results

### Optimizing Extraction and Immunoprecipitation of CLAG3 from Membranes

**Fig 1:**
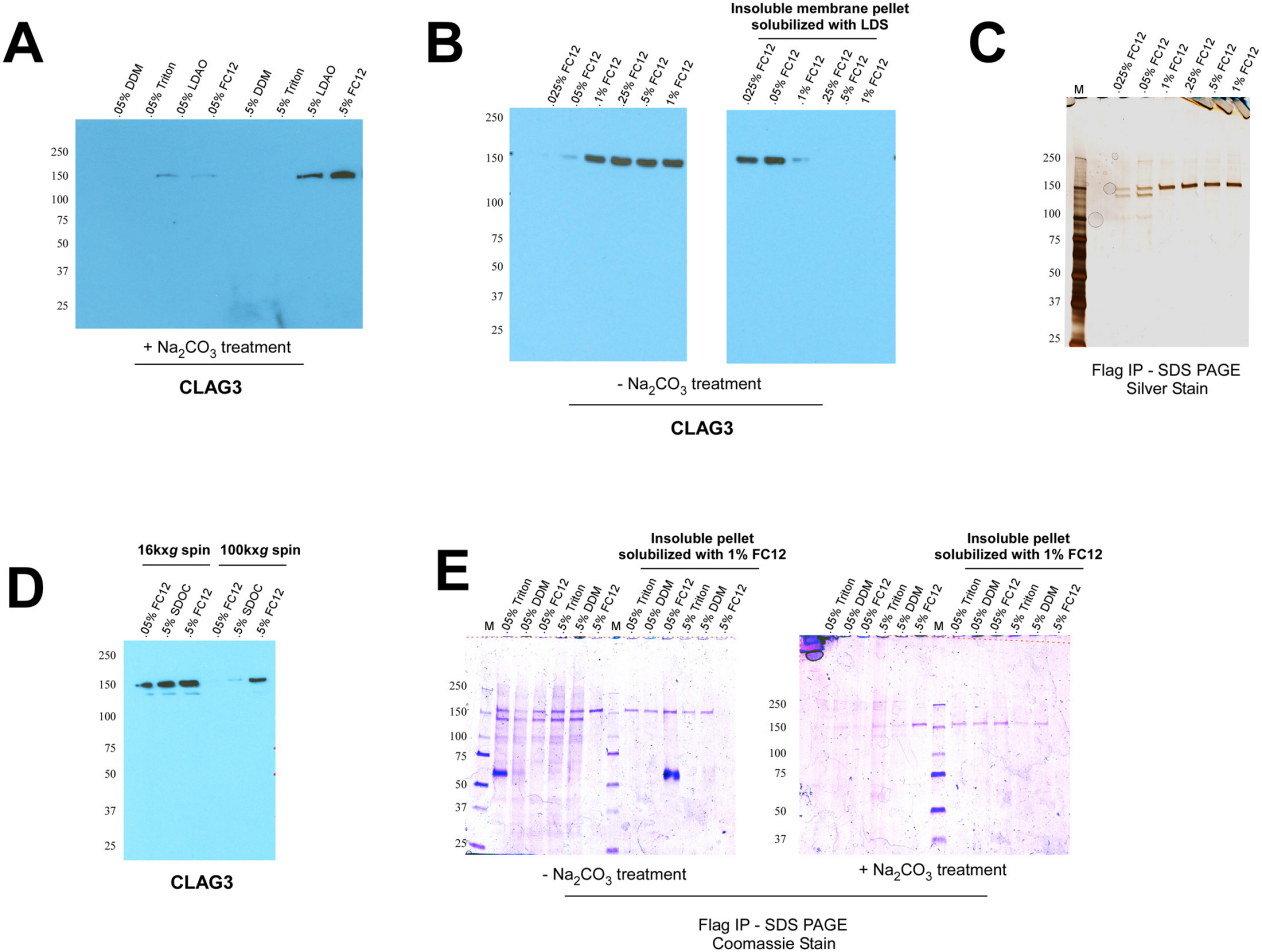
Solubilization and immunoprecipitation of membrane-associated CLAG3. (A) Western blot analysis shows that the zwitterionic detergents, lauryldimethyl amine oxide (LDAO) and fos-choline 12 (FC12), are able to solubilize integral CLAG3 at 0.5% (w/v) from sodium carbonate pH 11 (Na_2_CO_3_) treated trophozoite-infected erythrocyte membranes. (B) FC12 solubilizes total CLAG3 from (non-carbonate treated) membranes at concentrations 0.1% or above, leaving almost nothing behind in the insoluble membrane pellet. (LDS: lithium dodecyl sulfate). (C) Immunoprecipitation (IP) of CLAG3 from a previously described FLAG-tagged CLAG3 parasite (HB3^3REC^) results in the appearance of the expected 160kDa CLAG3 band at FC12 concentrations ≥ 0.1%, and two additional faint bands (150kDa and 100kDa) at FC12 concentrations ≤ 0.05% [8]. (D) Substantially more CLAG3 remains in the detergent-soluble supernatant following a 16,000x*g* spin to pellet insoluble material as compared to a traditional 100,000x*g* spin for all detergents tested (SDOC: sodium deoxycholate). (E) IP experiments were repeated incorporating the slower 16,000x*g* spin. All detergents tested (with the exception of 0.5% FC12) give rise to the aforementioned three bands in non-carbonate (-Na_2_CO_3_) treated membranes, but fail to do so in membranes treated with sodium carbonate (+Na_2_CO_3_). As evidenced by the right half of each gel, the only detergent able to fully solubilize CLAG3 from membranes (and leave nothing behind in the insoluble pellet) is 0.5% FC12. The prominent 60kDa band corresponds to heavy chain IgG.

It has previously been shown that CLAG3 exists both as a peripheral and integral membrane protein, with the integral fraction localizing to the infected erythrocyte membrane [8, 9]. Before performing immunoprecipitation experiments, I first needed to identify a non-denaturing detergent capable of solubilizing this integral pool of CLAG3. I found that the zwitterionic detergents, lauryldimethyl amine oxide (LDAO) and fos-choline 12 (FC12), efficiently solubilize CLAG3 at 0.5% w/v (but not 0.05%) from membranes treated with sodium carbonate to remove peripherally associated proteins (Fig 1A). Other commonly used detergents such as Triton X100 and n-Dodecyl β-D-maltoside (DDM) were unable to extract integral CLAG3 even at concentrations up to 2% (Fig 1A; **data not shown**).

I chose FC12 for further characterization as LDAO proved incompatible with downstream Native PAGE applications. At concentrations ≥ 0.1% FC12 fully extracts CLAG3 from membranes, leaving almost nothing behind in the insoluble membrane pellet (obtained after a 100,000x*g* spin) (Fig 1B). Next, to identify potential interacting partners I used a previously described FLAG-tagged CLAG3 parasite (HB3^3REC^) to immunoprecipitate (IP) CLAG3 after extraction with FC12 [8]. SDS PAGE analysis of IP eluates shows that CLAG3 immunoprecipitates as one band of expected size (160kDa) at concentrations ≥ 0.1% FC12. In contrast, at concentrations below 0.1% two additional faint bands of 150kDa and 100kDa can be observed (Fig 1C). The sizes of these bands are consistent with members of the RhopH complex, RHOPH2 and RHOPH3, which CLAG3 has previously been shown to interact with in the rhoptries of schizonts/merozoites [13, 14].

To bolster these findings, I next sought to improve my IP strategy. I hypothesized that the addition of detergents might create large micelles containing CLAG3 that are potentially lost during the 100,000x*g* spin that precedes immunoprecipitation. To test this, I used a slower speed of 16,000x*g* to pellet insoluble material and found substantially more CLAG3 remained in the supernatant (Fig 1D). Repeating IP experiments using this modified protocol, I found that the three aforementioned bands were now much more prominently visible after IP (Fig 1E).

Given the earlier observation that ≥ 0.1% FC12 was required to efficiently extract integral CLAG3 (Fig 1A and 1B), I hypothesized that the other detergents might be preferentially liberating peripheral pools of CLAG3. Consistent with this, sodium carbonate treatment of membrane fractions prior to IP (to remove peripheral proteins) resulted in the virtual disappearance of CLAG3 (and associated bands) for all detergents except 0.5% FC12 (Fig 1E). I next reasoned that if the aforementioned detergents were solubilizing only peripheral CLAG3, then integral CLAG3 should still remain in their insoluble membrane pellets. To test this, I used 1% FC12 to re-solubilize the resulting insoluble pellets and then performed a second FLAG IP. As expected, 1% FC12 allowed solubilization and recovery of the CLAG3 that had not liberated from the initial detergent treatments (Fig 1E). All together, these results indicate that ≥ 0.1% FC12 is capable of solubilizing integral CLAG3, whereas the other detergents (or ≤ .05% FC12, a concentration very close to its critical micelle concentration of 0.047% w/v) appear to extract peripherally associated CLAG3. The ability of ≥ 0.1% FC12 to extract integral CLAG3 however appears to come at the cost of disrupting potential protein-protein interactions.

### CLAG3 Participates in High Molecular Weight Membrane-associated Complexes

**Fig 2:**
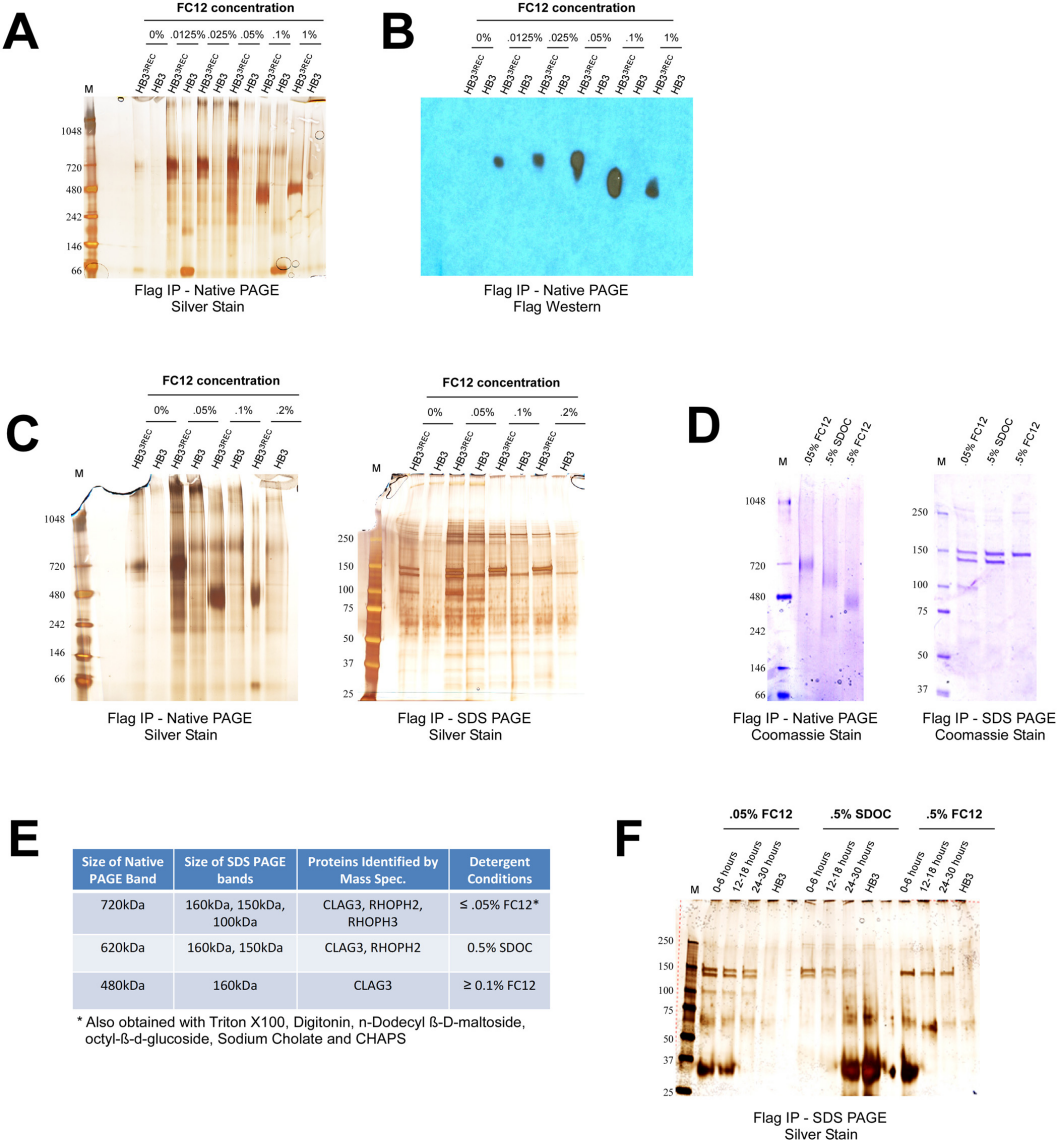
CLAG3 forms high molecular weight membrane-associated complexes. (A) Blue Native PAGE analysis of FLAG IP eluates obtained from a FLAG-tagged CLAG3 parasite (HB3^3REC^) and an isogenic untagged HB3 negative control [8]. Two distinct bands can be seen in HB3^3REC^ that are absent in HB3: a ~720kDa band for concentrations of FC12 ≤ 0.05% and a ~480kDa band for concentrations of FC12 ≥ 0.1%. (B) FLAG Western blot confirms that the 720kDa and 480kDa bands contain CLAG3. (C) SDS PAGE analysis shows that upon addition of SDS and 2-mercaptoethanol the 720kDa complex dissociates to three proteins (CLAG3, RHOPH2, and RHOPH3 as determined by mass spectrometry), while the 480kDa complex dissociates to only one protein (CLAG3). (D) Sodium deoxycholate (SDOC) at 0.5% (w/v) yields an intermediate ~620kDa Native PAGE band that contains CLAG3 and RHOPH2, but not RHOPH3 (as determined by SDS PAGE and mass spectrometry). (E) A summary of the mass spectrometry results for the indicated Native and SDS PAGE bands. (F) IP experiments on tightly synchronized parasites show that CLAG3, RHOPH2 and RHOPH3 appear to associate faithfully in early (0–6 hours post-invasion), mid-stage (12–16 hours post-invasion), or late rings/early trophozoites (24–30 hours post-invasion). The prominent 30kDa band corresponds to light chain IgG.

To determine if CLAG3 and its associated bands form a large complex, I next performed Blue Native PAGE analysis on IP eluates. Using an isogenic untagged HB3 parasite as a negative control, I found that CLAG3 participates in a ~720kDa complex at FC12 concentrations ≤ 0.05% and a ~480kDa complex at FC12 concentrations ≥ 0.1% (Fig 2A). Western blot analysis with FLAG antibody confirms that these bands contain CLAG3 (Fig 2B).

To identify the molecular components of these complexes, I divided the IP eluates into two fractions: one for Native PAGE and one for traditional SDS PAGE (addition of SDS and 2-mercaptoethanol should dissociate the complexes into their individual subunits). I found that under denaturing conditions the 720kDa complex disassociates to three bands (160kDa, 150kDa, and 100kDa), whereas the 480kDa complex dissociates to only one band (160kDa) (Fig 2C). To learn the identities of the proteins, mass spectrometry was performed on both the Native and SDS PAGE bands from the FLAG-tagged parasite, as well as the corresponding gel slice from the HB3 negative control (to account for false positives). Repeated analyses from at least two independent experiments identified the proteins in the 720kDa complex as CLAG3, RHOPH2 and RHOPH3; and the protein in the 480kDa complex as CLAG3 (Fig 2E; S1 Table).

To determine if additional CLAG3-containing complexes occur in the infected erythrocyte, I next repeated IP experiments using a more diverse array of detergents. While digitonin, CHAPS, sodium cholate, and octyl-β-d-glucoside gave rise to the 720kDa complex (**data not shown**), 0.5% sodium deoxycholate (SDOC) yielded an intermediate band of approximately 620kDa (Fig 2D). Mass spectrometry and SDS PAGE analysis shows that this complex contains CLAG3 and RHOPH2 (but not RHOPH3), indicating CLAG3 and RHOPH2 interact directly (Fig 2E). Subsequent IP experiments with varying salt (50mM-1000mM) and glycerol (0–15%) concentrations, and use of PSAC inhibitors (ISPA28, MBX2366, Dantrolene, Furosemide, and Phloridzin) failed to produce additional complexes or interacting proteins [7] (**data not shown**).

Since CLAG3, RHOPH2 and RHOPH3 are known to interact in the rhoptries of schizonts/merozoites, I was keen to confirm that they also associate earlier in the parasite lifecycle [13, 14]. Using tightly synchronized parasites, I found that the three proteins associate faithfully even in early rings and trophozoites where rhoptries are absent (Fig 2F).

### Cross-linking Studies Confirm the Existence of CLAG3 Complexes

**Fig 3:**
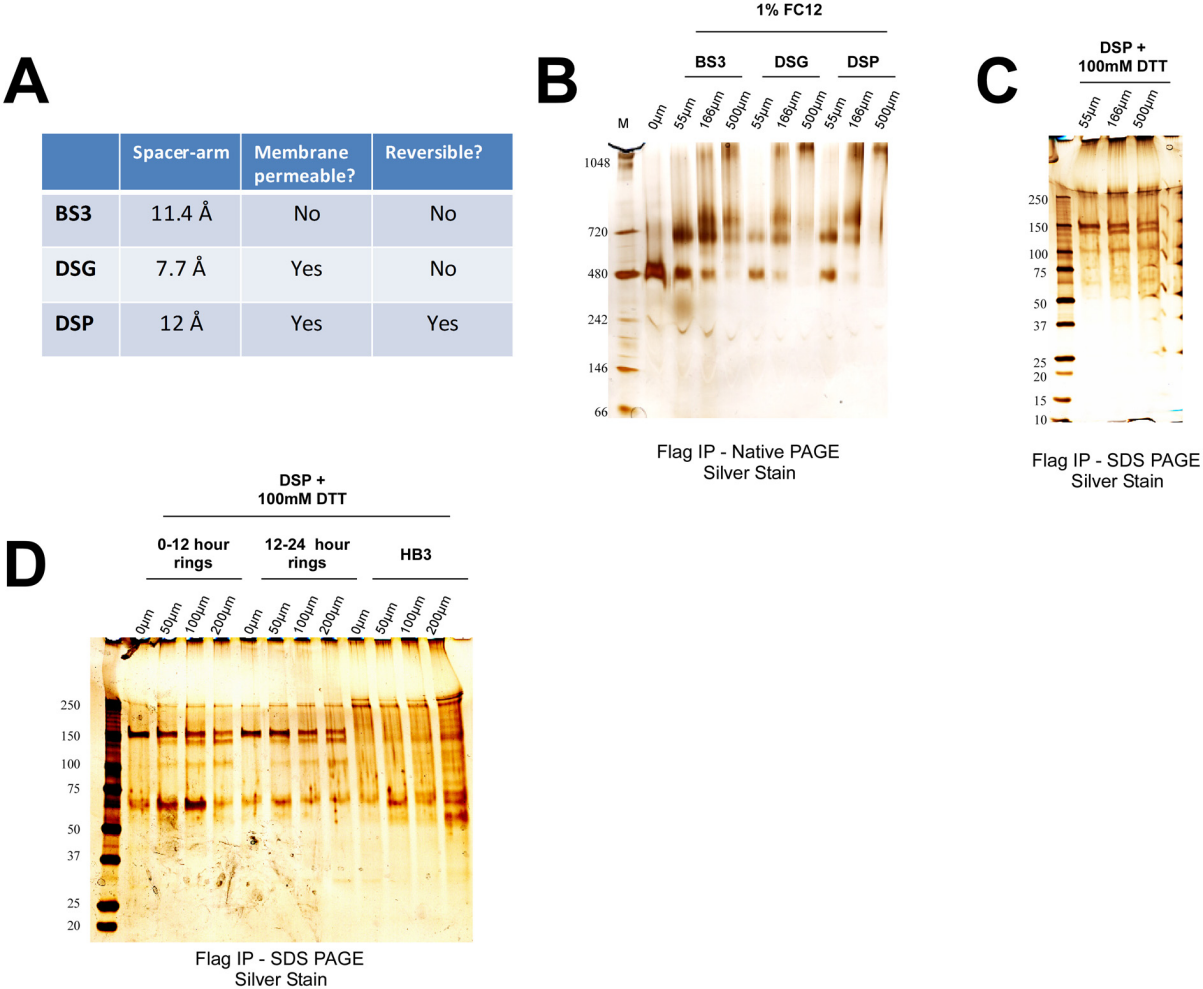
Cross-linking studies with NHS-esters confirm the existence of CLAG3 complexes. (A) The properties of the three different N-hydroxysulfosuccinimide esters (NHS-esters) used in this study: Bis(sulfosuccinimidyl) suberate (BS3), Disuccinimidyl glutarate (DSG), and Dithiobis (succinimidyl propionate) (DSP). (B) The 480kDa complex is observed with the use of 1% FC12, whereas addition of the three different NHS-esters results in the appearance of higher molecular weight complexes in a dose dependent manner (as determined by Native PAGE). (C) DSP cross-link was reversed with the addition of 100mM Dithiothreitol (DTT) and bands separated on SDS PAGE. The three prominent bands observed are consistent in size with CLAG3, RHOPH2, and RHOPH3. The band near 250kDa was subsequently found to also be present in HB3 negative control parasites (see next panel). (D) IP experiments on tightly synchronized parasites obtained without enrichment and cross-linked with DSP demonstrate no appreciable differences in the association CLAG3, RHOPH2, and RHOPH3 in early (0–12 hour) or late (12–24 hour) rings (when PSAC is inactive and active, respectively). HB3 was used as a negative control.

As independent confirmation of these results, and to identify additional interacting partners, I next performed cross-linking experiments to stabilize potentially labile/transient protein-protein interactions. I used varying concentrations of three different amine-reactive N-hydroxysulfosuccinimide esters (NHS-esters) to cross-link proteins in hypotonically lysed cells (Fig 3A). In samples that were not cross-linked, solubilization with 1% FC12 yields only the 480kDa Native PAGE band (Fig 3B). However, upon addition of increasing amounts of cross-linker, two larger complexes similar in size to the 620kDa and 720kDa bands begin to appear (Fig 3B).

To determine the molecular constituents of these complexes, I reversed the cross-link for DSP and ran the products using SDS PAGE (Fig 3C). As before, bands consistent with the sizes of CLAG3, RHOPH2 and RHOPH3 were the only prominent bands visible after silver staining. These findings suggest that 1% FC12 breaks the interaction of CLAG3 with RHOPH2 and RHOPH3 but that self-association of CLAG3 in the 480kDa complex is not disrupted.

To test whether these protein complexes might be present in parasitized erythrocyte stages when PSAC is inactive, I performed a similar cross-linking study in early and late ring-infected erythrocytes. Consistent with earlier findings, I did not observe any difference in the association of the three proteins in 0–12 hour ring-infected erythrocytes (when PSAC activity is not detected) as compared to 12–24 ring-infected erythrocytes (when PSAC activity is present) [5] (Fig 3D).

### The 480kDa Complex Composed of CLAG3 Localizes to the Erythrocyte Surface

**Fig 4:**
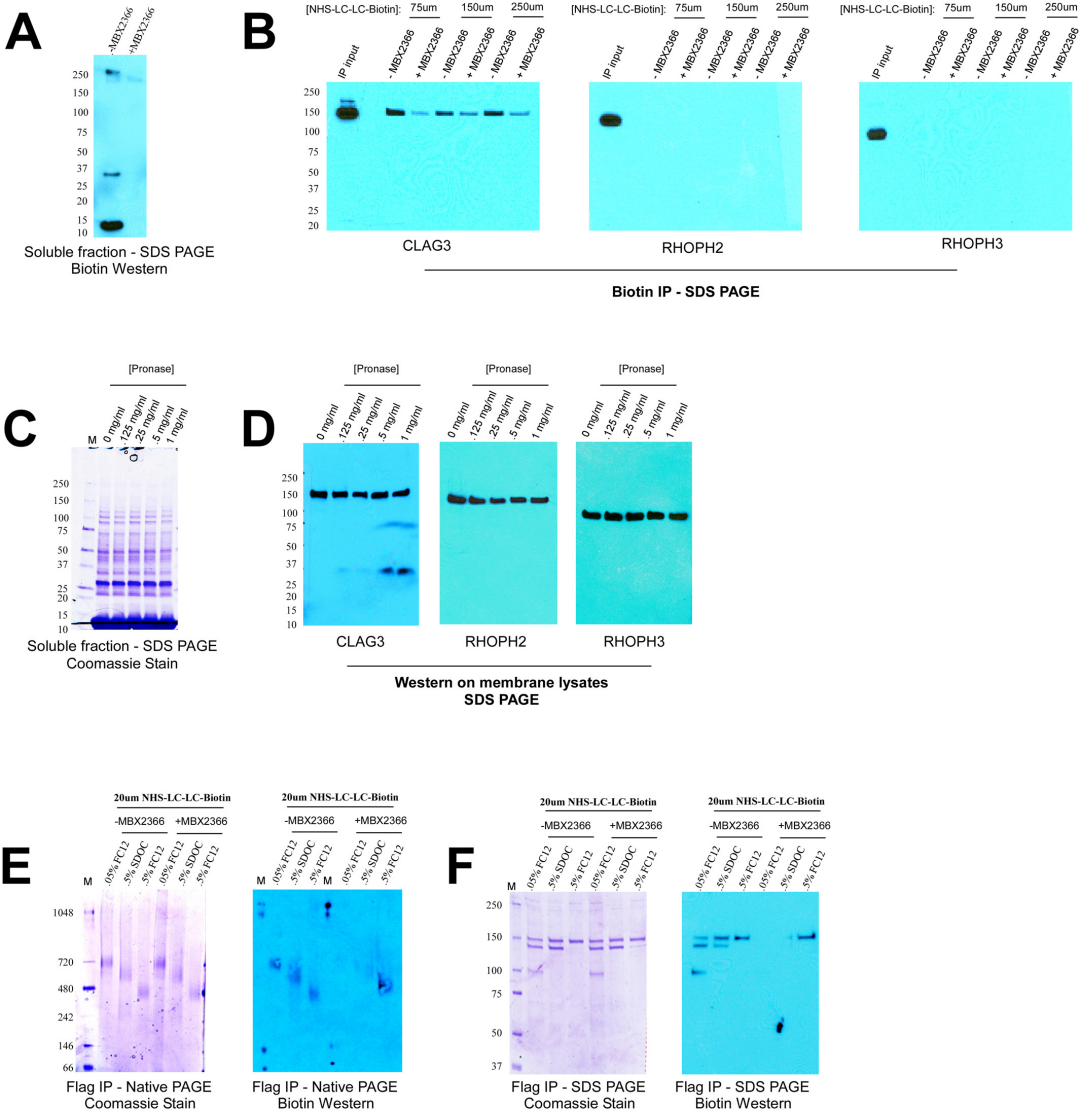
The 480kDa complex composed of CLAG3 localizes to the erythrocyte membrane. (A) Incubation of infected erythrocytes with extracellular NHS-LC-LC-Biotin results in labeling of intracellular proteins due to traversal through PSAC, which is prevented in the presence of PSAC inhibitor, MBX2366. (B) Membranes of biotin-labeled cells were solubilized with 1% FC12 and subjected to biotin IP, followed by Western blot for the indicated proteins. As indicated by the IP inputs, 1% FC12 is able to equally solubilize CLAG3, RHOPH2 and RHOPH3. However, only CLAG3 is found in the biotin IP eluate indicating localization to the erythrocyte surface. (C) Treatment with extracellular pronase-E does not result in the cleavage of intracellular proteins. (D) Extracellular pronase-E results in the appearance of a cleavage fragment for CLAG3, but not RHOPH2 and RHOPH3, in a dose-dependent fashion. C-terminal antibodies were used for all three proteins. (E) Extracellular biotin labeling results in labeling of the 480kDa complex in the presence of MBX2366 indicating that this complex localizes to the erythrocyte membrane. (F) Consistent with the above results, Western blot analysis with biotin antibody shows that only the CLAG3 obtained with 0.5% FC12 (corresponding to the 480kDa complex) labels with biotin in the presence of MBX2366.

Given their interaction with CLAG3 and thus possible role in channel function, I was next interested in further characterizing RHOPH2 and RHOPH3. To determine if these proteins localize to the erythrocyte surface, I performed extracellular biotin labeling experiments. It has previously been shown that NHS-LC-LC-Biotin enters infected erythrocytes via PSAC [6, 20]. Therefore, to prevent intracellular labeling of proteins I used the potent PSAC inhibitor MBX2366 to block PSAC activity during the labeling reaction [7] (Fig 4A). The membranes of biotinylated cells were then solubilized with 1% FC12, followed by biotin IP and Western blot for CLAG3, RHOPH2 and RHOPH3. Here, proteins that label strongly with biotin are immunoprecipitated and thus should be detectable by Western blot. As indicated by similar levels in the IP input, all three proteins are efficiently solubilized with 1% FC12 whereas only CLAG3 is detectable after biotin IP (Fig 4B). These results indicate that unlike RHOPH2 and RHOPH3, CLAG3 localizes to the erythrocyte surface with exposed residues (Fig 4B). Consistent with this, extracellular pronase-E treatment of intact cells results in the appearance of a cleavage product for CLAG3, but not for RHOPH2 and RHOPH3 (C-terminus antibodies were used for all three proteins) (Fig 4C and 4D). Together, these data provide strong evidence that CLAG3 is the only member of complex to definitively reside at the host membrane.

I next sought to determine which of the identified Native PAGE complexes localize to the erythrocyte surface. To achieve this, I performed a reciprocal IP experiment—biotin labeled cell membranes were immunoprecipitated with FLAG antibody, separated on Native PAGE and then probed by Western blot for biotin. As expected, all three complexes show biotin labeling in the absence of MBX2366, due to traversal of the label into the cell (Fig 4E). However, upon PSAC blockage only the 480kDa complex labels with biotin, indicating it localizes at the erythrocyte membrane (Fig 4E). Consistent with these results, SDS PAGE analysis shows that only the CLAG3 obtained with 0.5% FC12 (corresponding to the 480kDa complex), but not with 0.05% FC12 or 0.5% SDOC (corresponding to the 720kDa and 620kDa complexes, respectively), labels efficiently with biotin (Fig 4F). These results reinforce the model that only 0.5% FC12 is able to sufficiently solubilize integral CLAG3 whereas other detergents (such as 0.5% SDOC or 0.05% FC12) appear to preferentially extract peripheral CLAG3.

## Discussion

While the contribution of CLAG3 to PSAC function is firmly established, exactly how it contributes has remained elusive. It is possible that CLAG3 works *in trans* to activate host quiescent channels, or it might form the channel itself, with or without the help of other host/parasite proteins [21–26]. To answer these questions, an agnostic biochemical approach was taken in this study.

Experiments using diverse detergents revealed the presence of three different membrane-associated complexes containing CLAG3 (480kDa, 620kDa, and 720kDa). These complexes might either represent distinct entities or simply detergent-induced breakdown products of a larger complex. It is interesting to note that the CLAG3 from the smallest complex (480kDa) labels with biotin whereas the CLAG3 from the 620kDa and 720kDa complexes does not. These data suggest that the 480kDa complex contains integral CLAG3 whereas the 620kDa and 720kDa complexes contain peripheral CLAG3. Intriguingly, previous studies have reported this peculiar property of CLAG3 to exist both as a peripheral and integral membrane protein [8, 9]. The significance of these differing pools of CLAG3 (and associated complexes) currently remains a mystery.

One possibility is that the 480kDa complex represents the mature channel and the other larger complexes represent trafficking intermediates (or unassembled channels). Here, RHOPH2 and RHOPH3 possibly deliver CLAG3 to the host membrane and disassociate upon insertion of the 480kDa channel complex. In fact, following invasion members of the RhopH complex can be found at the parasitophorous vacuole membrane and Maurer’s cleft, organelles associated with trafficking [15, 16]. Another possibility is that the 480kDa complex represents the integral subunit (ie the pore) and that the 620kDa/700kDa complexes form the intracellular face of the channel. Such a model is consistent with previous work indicating that an intracellular component of PSAC is important for channel function [27]. Whether the 480kDa complex constitutes a breakdown product of an even larger complex, or instead represents the stable pore of the mature channel—similar to that of other channels [28–31]—will require further experimentation with even less denaturing conditions.

While far from conclusive, the data presented here nonetheless provide possible stoichiometries for the observed complexes (Fig 2E). For instance, the most parsimonious explanation for the ~620kDa complex is that it is composed of two molecules of CLAG3 (160kDa) and two molecules of RHOPH2 (150kDa). Accordingly, the ~720kDa complex would then simply consist of one additional molecule of RHOPH3 (100kDa). Consistent with this model, CLAG3 and RHOPH2 from these complexes stain with equal intensity in SDS PAGE, whereas RHOPH3 stains consistently lighter than the two (Figs 1C, 2D and 4F). Finally, the ~480kDa complex represents either a trimerized version of 160kDa CLAG3 or more likely a dimerized version that runs slow (in Native PAGE) due to associated lipids—a phenomenon reported for other integral membrane proteins [32–34]. Quantitative mass spectrometry experiments are currently underway in order to confirm whether these stoichiometries in fact hold true.

An outstanding question that remains is what exact role RHOPH2 and RHOPH3 play, if any, in PSAC function. It is noteworthy that neither protein could be genetically knocked out in large-scale experiments suggesting they have an essential role in the parasite [17]. Here I present evidence that they interact with CLAG3 and that this interaction persists in the newly parasitized erythrocyte. It is thus possible that RHOPH2 and RHOPH3 contribute directly to channel function or, as described above, they may act as chaperones for CLAG3 (and/or other yet unidentified PSAC proteins) to help indirectly in channel formation. Further studies, particularly with epitope-tagged RHOPH2 and RHOPH3 parasites will be required to determine whether they play any role in PSAC function and/or formation.

A final mystery is as to why it takes many hours after invasion for PSAC to become active. This is particularly curious as all three proteins are injected almost immediately upon invasion. The possibility has been put forth that trafficking through the Plasmodium Translocon of Exported Proteins (PTEX) is required for activation of PSAC [35]. However, CLAG3 localizes to the erythrocyte membrane even in the absence of PTEX, suggesting trafficking of another protein is required [35]. I surmised that RHOPH2 and/or RHOPH3 might represent this protein. However, I found that all three proteins associated even in early rings where PSAC is inactive. Therefore, it is possible that another trafficked protein is required for the formation and/or activation of PSAC.

Here, I have presented evidence that CLAG3 participates in multiple high molecular weight complexes, one of which localizes to the erythrocyte surface. This 480kDa complex, likely composed of an oligomerized version of 160kDa CLAG3, is integral to the erythrocyte membrane and thus serves as an attractive candidate as the possible pore of PSAC. Consistent with this, CLAG3 is known to contain at least one amphipathic transmembrane domain which resembles that of a water-filled pore [19]. Whether additional functional subunits are required for full channel activity and precisely how they associate to form the mature channel all remain open questions. I hope these findings provide a useful starting point for deciphering how this unusual channel functions in the malaria infected erythrocyte.

## Supporting Information

S1 Table Summary of mass spectrometry results. Each tab represents an independent mass spectrometry experiment analyzed with PEAKS v. 7.5.
(XLS)
